# A neurobiological evaluation of soft touch training for patients with skin-picking disorder

**DOI:** 10.1016/j.nicl.2022.103254

**Published:** 2022-11-03

**Authors:** Anne Schienle, Carina Schlintl, Albert Wabnegger

**Affiliations:** Clinical Psychology, University of Graz, BioTechMed Graz, Austria

**Keywords:** Skin-picking disorder, Soft touch training, Functional magnetic resonance imaging, App-assisted training

## Abstract

**Background:**

Individuals with skin-picking disorder (SPD) display reduced neural sensitivity to slow/soft touch. This functional magnetic resonance imaging (fMRI) study investigated the effects of Soft Touch Training (STT) on neural correlates of touch processing and SPD symptoms.

**Method:**

Females with a primary diagnosis of SPD (n = 57) were randomly assigned to receive either four weeks of STT (guided soft brushing of selected skin regions) or Progressive Muscle Relaxation (PMR) training (guided tensing and relaxing of selected muscle regions). The app-assisted intervention consisted of 15-minute daily training sessions at home. Before and after the four-week intervention, the two groups participated in a standardized tactile stimulation procedure during fMRI (affective vs nonaffective touch of the forearm). Intervention-based changes in subjective and neural responses to the tactile stimulation as well as SPD severity were compared between the groups.

**Results:**

STT increased the pleasantness ratings for (affective) touch administered during fMRI, which was accompanied by decreased activation in the parietal operculum (PO) and supramarginal gyrus (SMG), as well as increased PO-SMG connectivity. These findings possibly reflect normalized affective touch processing due to STT. Both interventions (STT and PMR) reduced SPD severity.

**Conclusions:**

This study demonstrated that a brief app-assisted touch training can change the experience of receiving touch from others and the associated brain activity and connectivity. Adaptations of the training relating to duration/ frequency of sessions might enhance its effectiveness.

## Highlights

• Patients with skin-picking disorder (SPD) display reduced neural sensitivity to affective/soft self-touch.
• Neurobiological evaluation of a new Soft Touch Training (STT) for SPD patients.
• STT increased the pleasantness ratings for (slow/soft) touch administered during fMRI.
• STT changed activity/ connectivity in the secondary somatosensory cortex.

## 1 Introduction

Skin-picking disorder (SPD) is a common mental disorder. Estimates of prevalence range from 1 to 5 % in the general population with females being more frequently affected than males (ratio: 3:1; American Psychiatric Association, 2013, Grant and Chamberlain, 2020).

The predominant symptom of SPD involves the repeated picking of one’s skin in areas such as the hands, arms, and face. The picking is mainly carried out with fingernails (or more rarely with tools such as tweezers and needles) (for a review see Grant et al., 2012). The consequences of compulsive skin-picking can be serious. It can lead to severe tissue damage and associated complications (e.g., infections). Some patients are covered with sores and scars, leading to disfigurement. In addition to physical injury, SPD causes clinically significant distress and impairment in important areas of functioning (APA, 2013).

Several lines of evidence point to altered touch processing in patients with SPD. First, studies on sensory over-responsivity have shown enhanced sensitivity to specific tactile stimuli in individuals who display body-focused repetitive behaviors (BFRBs, such as compulsive hair-pulling/ skin-picking; Falkenstein et al., 2018, Houghton et al., 2018, Houghton et al., 2019). These individuals overreact to weak tactile input; for example, the touch of ordinary textures of cloth such as a soft towel can cause discomfort.

Second, patients with SPD show atypical emotional responses to their skin picking. The picking is experienced as ‘soothing’, ‘satisfying’, and/or ‘tension- releasing/relaxing’ by most patients, even though the excessive skin manipulation leads to tissue damage (Schienle et al., 2020).

Third, studies using functional magnetic resonance imaging (fMRI) have indicated that people with SPD show atypical neural processing of affective touch. In one study (Schienle et al., 2018), participants were instructed to either caress or scratch a small area of skin on their arms. Compared to healthy controls, SPD patients showed reduced activity during gentle self-touch in frontal and primary/secondary somatosensory cortex regions.

In sum, these studies suggest differences in sensitivity to soft/ affective touch in individuals with SPD. It is however unclear if this altered sensitivity can be changed by means of a training and how this could improve symptoms of SPD.

The present study investigated a new training method for people with SPD, one that aims at altering the perceived pleasantness of affective touch, along with the associated brain activity. Brain regions involved in the processing of affective touch include the primary/secondary somatosensory cortex, the insula, the striatum, and prefrontal cortex regions (Olausson et al., 2002, Morrison, 2016). An activation likelihood estimate (ALE) meta‐analysis using fMRI detected three ‘affective-touch clusters’ which encompass the posterior insula and the right/ left parietal operculum (Morrison, 2016). Based on these activation clusters, it was possible to differentiate between affective and nonaffective touch. In the current study, the neural responses of participants to affective and nonaffective touch were recorded pre and post the newly developed training method: ‘Soft Touch Training’ (STT). STT was carried out at home. A smartphone application was used to deliver auditory instructions that guided self-touch of selected skin regions, using a soft brush (e.g., left/ right forearm, face). The control intervention was Progressive Muscle Relaxation (PMR) training (guided tensing and relaxing of selected muscle regions, such as left/ right forearm, face). Participants were asked to practice daily (approximately 15 min) for four weeks, and rate their experience of the daily training sessions via the app (e.g., affective state, urge to pick their skin).

Before and after the four-week training, an fMRI session was conducted. A standardized tactile stimulation procedure was used to compare emotional and neural responses to affective vs nonaffective touch between the two groups (slow vs fast brushing of patients’ forearms by an experimenter). According to the preregistration (https://www.drks.de/drks; ID = DRKS00022123), it was expected that participants in the STT group would rate affective touch as being more pleasant and would display reduced symptom severity (as assessed with disorder-specific questionnaires) after the training. This should be associated with activation changes in the insula, the somatosensory cortex, the striatum, the prefrontal cortex, and the thalamus.

## 2 Method

### 2.1 Participants

Fifty-seven female patients with a primary DSM-5 diagnosis (APA, 2013) of skin- picking disorder were randomly allocated (random number table) to Soft Touch Training (STT) or Progressive Muscle Relaxation (PMR). PMR was chosen as an active control condition with a comparable duration and frequency of instructions/training sessions targeting the same body regions as STT.

Exclusion criteria (as checked with an online screening) were diagnoses of major depression with severe symptoms, substance abuse/dependence, psychosis, and dermatological conditions (e.g., scabies, psoriasis, atopic dermatitis). One participant abandoned the PMR training after one day and was therefore excluded from statistical analysis. The two groups (STT: n = 30; PMR: n = 26) did not differ in mean age, symptom severity/ duration, and comorbidity (see Table 1).

**Table 1:** Characteristics of the two training groups.

	**STT (n = 30)** **M (SD)**	**PMR (n = 26)** **M (SD)**	**t(df)**	**p**
**Age (years)**	26.27 (9.41)	24.23 (2.88)	1.06 (54)	0.29
**Symptom duration (years)**	14.51 (7.62)	12.35 (5.67)	1.19 (54)	0.24
**Lifetime/Current comorbidity**	**Percent**	**Percent**	**Chi^2^ (df)**	**p**
Anxiety disorders*	33 %/27 %	46 %/39 %	0.96/ 0.89	0.33/0.35
**Eating disorders**	17 %/ 10 %	12 %/ 0 %	0.29/ 2.74	0.58/0.10
**Obsessive-compulsive disorder**	7 %/ 3 %	4 %/ 4 %	0.22/ 0.01	0.64/0.92
**Depression**	33 %/ 3 %	23 %/ 8 %	0.72/ 0.53	0.40/0.47

Abbreviations: STT, Soft Touch Training; PMR, Progressive Muscle Relaxation; M, mean; SD, standard deviation.

*Generalized anxiety disorder or panic disorder.

The study complied with all relevant ethical guidelines and regulations involving human participants (Code of Ethics of the World Medical Association; Declaration of Helsinki) and was approved by the ethics committee of the University of Graz, Austria (GZ 39/29/26 ex 2018/19). All participants provided informed consent before participating. This study was preregistered on the German Clinical Trials Register (DRKS00022123, June 8th, 2020).

The sample was restricted to females because of gender differences concerning the prevalence of SPD and affective touch processing (American Psychiatric Association, 2013, Grant and Chamberlain, 2020, Jönsson et al., 2017).

### 2.2 Questionnaires

The participants completed the following questionnaires:

a) The Skin-Picking Scale revised (SPS-R; Gallinat et al., 2016; Cronbach’s α (present sample) = 0.82) is a self-report questionnaire to assess the severity of skin- picking symptoms during the last week. The scale contains eight items covering the following domains: (1) frequency of the urge to pick one’s skin, (2) intensity of the urge to pick, (3) time spent picking, (4) control over picking, (5) functional impairment, (6) emotional distress, (7) avoidance behavior, (8) skin damage due to picking. Each item is rated on a 5-point scale from 0 (none) to 4 (extreme). We computed a total score.
b) The short version of Skin Picking Impact Scale (SPIS-S; Snorrason et al., 2013 α = 0.70) assesses the psychosocial impact of SPD symptoms during the last week with 4 items (e.g., “I feel unattractive because of my skin picking”; “My relationships have suffered because of my skin picking”). Items are rated on a 5-point scale from 1 (not) to 5 (very much). A total score is obtained by adding up the items.

### 2.3 App-assisted training

Participants received auditory instructions (via a smartphone app) that guided either soft brushing of the skin (STT) or muscle tensing/relaxing (PMR). Both interventions targeted the same body regions: left/ right forearm, left/ right upper arm, left/ right upper leg, left/ right cheek, lips. All participants were introduced to the training and the functioning of the app (and the brush) during the initial diagnostic session.

The STT instruction started as follows:

*Set aside about 15 min to complete this exercise. Find a quiet place free from distractions. Wear clothes that allow the touching of your legs and arms. Adjust the temperature of the room so that you feel comfortable. Recline in a comfortable chair or lie on your bed. Remove glasses or contacts and do not forget to remove your shoes. Take a few slow even breaths, breath in……and out… in …. and…. out.*

*Now take the brush with your right hand and start brushing your left forearm…..up…………..and down…………. up and down…focus your attention on your left forearm. If you like, you can close your eyes. Feel the caressing of your skin.* (The instruction controlled the velocity of the brushing).

Before each training session, the participants rated their current affective state via the app: How tense are you right now? How pleasant do you feel? How intense is your urge to pick your skin? (1 = not tense/ not pleasant/ no urge; 9 = very tense/ very pleasant/ very strong urge to pick one’s skin),

Directly after each training session, participants answered the same questions again (How tense are you right now?, How pleasant do you feel?, How intense is your urge to pick your skin?). At the end of the intervention, participants were asked to rate the perceived effectiveness of the training (1 = not effective; 9 = very effective).

### 2.4 Procedure

Individuals were recruited via the outpatient clinic of the university and social media. After an online screening (checking for inclusion/ exclusion criteria; disorder-specific questionnaires), eligible participants were invited to a diagnostic session. They were interviewed with a standardized diagnostic interview for mental disorders (Margraf, 1994) with additional questions concerning skin-picking symptoms according to DSM-5 (based on the Yale-Brown Obsessive-Compulsive Scale Modified for Neurotic Excoriation (NE-YBOCS). At the end of the diagnostic session, the training procedure was explained as well as the use of the smartphone app. Subsequently, the first fMRI session (affective/nonaffective touch) was conducted. After four weeks of training, the participants were invited to the second fMRI session. Finally, the disorder-specific questionnaires were filled out again.

### 2.5 fMRI sessions with tactile stimulation

During the fMRI sessions, a well-validated affective touch procedure was used (Taneja et al., 2019). A trained female research assistant administered affective touch and non-affective touch (as the control condition) via a hand-held soft boar bristle brush. Affective touch had a velocity of 3 cm/s and an approximate indentation force of 0.3 N on the left forearm (stroking in proximal to distal direction, 8 cm region). This type of brushing is optimal to activate low-threshold unmyelinated mechanoreceptors (C-tactile afferents). It mimics human caressing and is perceived as particularly pleasant (Löken et al., 2009, Taneja et al., 2019). Nonaffective touch (fast brushing) had a velocity of 30 cm/s.

The two brushing conditions (slow/fast) lasted for 6 s and were guided by a metronome via headphones. Each condition was repeated 12 times interspersed by rest blocks (no brushing) lasting for 12 s. The sequence of the brushing conditions was randomized. After each condition, the participants verbally rated their emotional state (valence, arousal) on a 9-point scale (9 = very pleasant, very aroused) and the urge to pick their skin (9 = maximal urge). A first signal tone (presented for 2 s) after each condition indicated to open the eyes and respond to the visually presented rating scales (12 s). A second signal tone (2 s) indicated to close the eyes for the subsequent brushing condition.

### 2.6 fMRI recording and analysis

The MRI session was conducted with a 3 T scanner (Vida, Siemens, Erlangen, Germany) with a 64-channel head coil. Functional runs were acquired using a T2*- weighted multiband *EPI* protocol (number of slices: 58, interleaved, flip angle = 82°, slice thickness: 2.5 mm; slice spacing: 3 mm; TE = 0.03; TR = 1800 ms; multi-band accel. factor = 2; acquisition matrix: 88; in-plane resolution = 2.5 × 2.5 × 2.5 mm). Structural images were obtained using a T1-weighted MPRAGE sequence (voxel size: 1 × 1 × 1 mm; acquisition matrix: 224, slice thickness: 1 mm, TE = 0.00236, TR = 1600 ms; flip angle = 9°). All analyses were conducted with SPM12 (version: 7487; Wellcome Department of Cognitive Neurology, London) and the generalized PsychoPhysiological Interactions toolbox (gPPI; McLaren et al., 2012).

Functional images of both sessions were first realigned and unwarped by registering images to the first image with a 2nd Degree B-Spline interpolation. Subsequently, T1-weighted structural images from both sessions were longitudinally registered (pairwise). The resulting average structural images were segmented into gray matter, white matter, and cerebrospinal fluid, which were further used to create a skull-stripped image. Realigned images were co-registered to the skull- stripped image using the normalized mutual function. Segmented white and gray matter images were used to create a study-specific template with the shoot toolbox implemented into SPM12. The resulting deformation fields were used to bring the realigned functional images into MNI Space (voxel size: 3 mm isotropic), which were finally smoothed with a Gaussian full-width at half maximum (FWHM) of 8 mm.

For the first-level analyses, the following regressors were included for both sessions (before and after four weeks of training) in the same design matrix (affective brushing, nonaffective brushing, rating scale). Additionally, the six motion parameters were introduced as regressors of no interest. Event-related time series were convolved with the canonical hemodynamic response function and the contrast of interest (after_affective - after_nonaffective)-(before_affective - before_nonaffective) was built. Data were high-pass filtered (175 s) and serial correlations were accounted for by using an autoregressive AR(1) model.

To investigate connectivity patterns, the gPPI approach was used. A 6-mm sphere built around the activation peak found in the interaction contrast (seeds: supramarginal gyrus: MNI-coordinates: 60,-24,24 and parietal operculum: MNI- coordinates: 60,-24,21). The extracted first eigenvariate for both seeds was used as a regressor in two separate GLMs (general linear models).

### 2.7 Statistical analysis

a) *Self-reports assessed via the smartphone app*: Mixed-model analyses of variance (ANOVAs) were conducted to compare the two GROUPS (STT, PMR) before vs after a daily training session (factor: TIME) according to experienced tension, pleasantness, and urge to pick one’s skin. The daily ratings had been averaged across the 28-day training period (M_before, M_after). Moreover, effectiveness ratings for the training were compared between the groups via a *t*-test.
b) *Self-reports concerning touch experience during the fMRI experiment*: A mixed-model ANOVA tested the effects of GROUP (STT, PMR), TOUCH (Affective, Nonaffective), and TIME (before, after the four-week training) on self-reports for valence, arousal and urge to pick one’s skin. The 12 ratings for the Affective-Touch conditions as well as the 12 ratings for the Nonaffective-Touch conditions were averaged.
c) Disorder-specific questionnaires: A mixed-model ANOVA tested the effects of GROUP (STT, PMR), and TIME (before, and after the four-week training) on the scores obtained on the questionnaires.

The analyses of the subjective data were performed with SPSS (version 28.0).

We report partial eta squared as an effect size measure.

d) *Brain imaging data:* For the second-level analysis, the contrast of interest was compared between groups (STT vs PMR) using a two-sample *t*-test. To restrict analyses to signals deriving from gray matter, an explicit mask built from all individual gray matter images was applied (threshold: 0.2). We computed region-of-interest (ROI) analyses for the left and right insula, the somatosensory cortex (supramarginal gyrus, and parietal operculum), the striatum, the prefrontal cortex, and the thalamus. For inferences, we considered voxel peaks as statistically significant when p corrected for family-wise error (FWE) was below 0.05. Unilateral masks for the ROI analyses were taken from the Harvard- Oxford probability atlas (threshold: 25 %).

## 3 Results

### 3.1 App ratings

The training lasted for 28 days. On average, participants used the app on 22 training days (SD = 6.93).

*Tension:* The main effect TIME (F(1,54) = 94.92, p <.001, *η2p* =.64) was statistically significant. On average, participants felt less tense after a training session (M = 3.41, SD = 0.94) than before (M = 4.15, SD = 1.08). The main effect GROUP (F(1,54) = 0.59, p =.447, *η2p* =.01) and the interaction GROUP × TIME (F(1,54) = 2.01, p =.162, *η2p* =.04) were not significant.

*Pleasantness:* The main effect TIME (F(1,54) = 36.69, p <.001, *η2p* =.41) was statistically significant. On average, participants felt more pleasant after a training session (M = 5.58, SD = 0.95) than before (M = 5.16, SD = 0.91). None of the other effects reached statistical significance (GROUP: F(1,54) = 2.45, p =.123, *η2p* =.04, TIME × GROUP: F(1,54) = 0.60, p =.443, *η2p* =.01).

*Urge to pick one’s skin:* The main effect TIME (F(1,54) = 117.12, p <.001, *η2p* =.68) and the interaction TIME X GROUP (F(1,54) = 8.37, p =.005, *η2p* =.13) were statistically significant. The urge to pick decreased after a training session in both groups. The decrease was more pronounced after PMR (Mdiff = 1.00, SDdiff = 0.58) compared to STT (Mdiff = 0.58, SDdiff = 0.53). The main effect GROUP did not reach statistical significance (F(1,54) = 0.11, p =.747, *η2p* =.002).

*Perceived effectiveness of the training*: The PMR group (M = 4.85, SD = 0.89) and the STT group (M = 4.77, SD = 1.40) did not differ in their effectiveness ratings for the training (t(49.79) = 0.27; p =.75).

### 3.2 Touch experience during the MRI experiment

*Arousal:* The main effects for TOUCH (F(1,54) = 36.49, p <.001, *η2p* =.40) and TIME (F(1,54) = 5.63, p =.02, *η2p* =.09) were statistically significant. Nonaffective touch (M = 4.05, SD = 1.38) was rated as more arousing than affective touch (M = 3.10, SD = 1.01). Before the four-week training, the arousal ratings were higher (M = 3.79, SD = 1.17) than after the training (M = 3.37, SD = 1.28). None of the other effects reached statistical significance (Supplementary Table S1).

*Valence:* The main effect TOUCH (F(1,54) = 95.53, p <.001, *η2p* =.64) and the interaction TIME X GROUP (F(1,54) = 16.60, p <.001, *η2p* =.24) were statistically significant. Affective touch (M = 6.31, SD = 1.48) was rated as more positive compared to nonaffective touch (M = 3.91, SD = 1.32). In the STT group, touch was rated as more positive after the four-week training (Mdiff = 0.57, SDdiff = 1.10). The PMR group showed the opposite trend (Mdiff = -0.43, SDdiff = 0.65).

Separate analyses for the two touch conditions indicated that in the STT group, affective touch was rated as more positive after the four-week training (Mdiff = 0.72, Sddiff = 1.35; t(29) = 2.94, p =.006), whereas valence ratings for nonaffective touch did not change (Mdiff = 0.42, SDdiff = 0.1.44; t(29) = 1.61, p =.119). In the PMR group, affective touch (Mdiff = 0.43, SDdiff = 0.97; t(25) = -2.28, p =.031) and nonaffective touch (Mdiff = 0.43, SDdiff = 0.85; t(25) = -2.60, p =.016) were rated as less positive after the training.

*Urge-to-pick one’s skin:* The main effect TOUCH (F(1,54) = 21.70, p <.001, η2p =.29) was statistically significant. Nonaffective touch was associated with a greater urge to pick the skin (M = 3.74, SD = 1.55) than affective touch (M = 2.97, SD = 1.21). None of the other effects reached statistical significance (all p >.05; see Supplementary Table S1).

### 3.3 Questionnaires

*SPS-R:* The ANOVA revealed a significant main effect for Time (F(1,52) = 28.52, p <.001, *η2p* =.35). After the training, the participants indicated a lower symptom severity (Mbefore = 16.20, SDbefore = 2.93; Mafter = 13.46, SDafter = 4.13). The main effect group and the interaction effect Group × Time were not significant (p >.05).

*SPIS-S:* The ANOVA revealed a significant main effect for Time (F(1,52) = 7.32, p =.009, *η2p* =.12). The training reduced the negative psychosocial impact of skin- picking (Mbefore = 14.50, SDbefore = 3.32; Mafter = 13.69, SDafter = 3.49). The main effect Group and the interaction effect Group × Time were not significant (p >.05).

## 4 Brain activity

### 4.1 Between-group effects

*Pre-training*: The two groups (STT, PMR) did not differ in their brain activation before the training (no significant whole-brain effects and ROI effects).

**Fig. 1:**
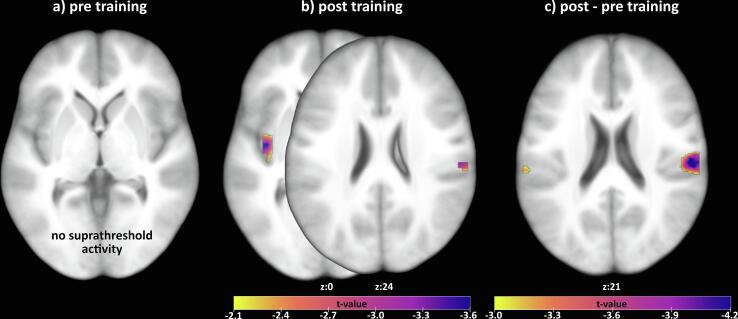
Group comparisons (PMR vs STT) for the contrast Slow vs Fast Touch.

*Changes in brain activation due to the training:* Relative to the PMR group, the STT group showed reductions in activity in the right parietal operculum (MNI coordinates: 60,-24,21, t = 4.27, p(FWE) = 0.003) and the right supramarginal gyrus (MNI coordinates: 60,-24,24, t = 4.25, p(FWE) = 0.009) (contrast: affective vs nonaffective touch: post vs pre-training; Fig. 1).

*Post-training:* Relative to the PMR group the STT group showed decreased activity in the left posterior insula (MNI coordinates: −39,-9,0, t = 3.66, p(FWE) = 0.032) and right supramarginal gyrus (MNI coordinates: 57, −21,24, t = 3.30, p(FWE) = 0.049).

All non-significant effects (p >.05) for the remaining ROIs can be found in Supplementary Table S2.

### 4.2 Within-group effects

*STT group:* Activation in the right parietal operculum (MNI coordinates: 60,-24,21, t = 3,97, p =.011), right supramarginal gyrus (60,-24,24, t = 4,37, p(FWR) = 0.006), and right posterior insula (36,-21,6; t = 3,39, p(FWE) = 0.041) decreased from the first to the second fMRI session.

*PMR group:* ROI activation did not change from the first to the second fMRI session.

All non-significant effects (p >.05) for the remaining ROIs can be found in Supplementary Table S2.

### 4.3 Changes in brain connectivity for regions of interest

After the training, the STT group (relative to the PMR group) showed increased functional connectivity (contrast: affective vs nonaffective touch) between the right supramarginal gyrus (seed) and the parietal operculum (ROI: MNI coordinates: 36,- 33,21, t = 3.30, p(FWE) = 0.043). The PMR group showed no changes in ROI connectivity after the training.

## 5 Discussion

This study investigated an app-assisted training method to improve affective touch processing in females with a diagnosis of SPD. Participants were randomly allocated to four weeks of at-home practice that included either Soft Touch Training (STT) or Progressive Muscle Relaxation (PMR). The evaluation of this new intervention was based on self-reports as well as neural indicators.

It was found that STT increased the perceived pleasantness of touch during the fMRI session, carried out after the end of the training. The STT group gave more positive valence ratings, particularly for the gentle brushing of their skin (affective touch). This subjective response from the STT group was accompanied by specific changes in brain activity and connectivity. Analysis of the neuroimaging data revealed STT-associated activation changes in regions of the secondary somatosensory cortex. Compared to PMR, STT reduced the activation during affective touch in the parietal operculum (PO) and the supramarginal gyrus (SMG).

Firstly, relevant functions of the PO and the SMG will be shortly reviewed, and then possible reasons for the reduced activation found in these areas after the STT in the current study will be discussed. The PO is a central brain hub for the processing of affective touch: An ALE *meta*-analysis by Morrison (2016) indicated that the PO has a high likelihood of being recruited during slow/gentle touch. Moreover, the PO is activated during discriminative touch, indicating its integrative role in both aspects of tactile information (discrimination and hedonic value of touch). In addition, studies on sensory attenuation (Blakemore et al., 1998, Limanowski et al., 2020, Shergill et al., 2013) have identified the role of the PO in differentiating between self-generated and externally generated somatosensory stimulation.

A similar function has been attributed to the SMG. A study by Boehme et al. (2019) indicated that the SMG was activated during touch from others (together with brain regions such as the insula, and the prefrontal cortex). In contrast, self-touch led to deactivation in the same regions (e.g., the insula, and prefrontal areas).

According to the ‘forward model’, the brain attenuates the perception of self-generated sensory stimuli to enhance the perception of external stimuli (e.g., Kilteni & Ehrsson, 2020). In a study by Schienle et al. (2018) patients with SPD showed pronounced hypoactivation during gentle self-touch (in somatosensory and frontal brain areas), when compared with healthy controls. This possibly reflects hyper-attenuation of self-generated tactile stimulation in those with SPD, which might go hand in hand with over-responsivity to external tactile stimulation. In line with this hypothesis, two recent studies (Falkenstein et al., 2018, Houghton et al., 2018) have shown that individuals who carry out body-focused repetitive behaviors (compulsive hair-pulling, skin-picking) tend to display heightened reactions to weak externally generated tactile stimuli. To sum up, the altered brain activity seen in participants after the STT in the current study (reduced activation in the PO and SMG areas), might thus reflect a kind of 'normalized’ processing of externally generated affective (and nonaffective) touch.

The connectivity analysis in the current study indicated that STT increased the functional coupling within the secondary somatosensory cortex. Participants in the STT group displayed stronger connectivity between the PO and the SMG compared to the PMR group. It has been shown that practicing specific tasks (e.g., finger tapping) increases functional connectivity in sensorimotor regions (Rogers et al., 2007), which very likely reflects more efficient processing; in contrast, patients with sensorimotor impairment after stroke exhibit reduced functional connectivity in the associated brain networks. In this way, the STT might have, by enhancing connectivity, also ‘normalized’ (non)affective touch processing in patients with SPD.

The app data showed that both STT and PMR positively influenced the affective state of the participants (less tension, more positive valence). Moreover, the urge to pick was reduced after the daily training sessions. The latter effect was even more pronounced in the PMR group. The majority of patients with SPD report that their skin-picking is elicited by negative affective states with elevated levels of tension or arousal (e.g., frustration, anger). The manipulation of the skin serves emotion regulation; the reduction of tension (Snorrason et al., 2010). Therefore, it is not surprising that a classical relaxation technique such as PMR would reduce the tension-related urge to pick. The present findings indicate that using relaxation training is a helpful approach to controlling temporary urges to pick one’s skin. Similarly, STT also had a relaxing effect in addition to the aspect of somatosensory training.

Finally, both interventions reduced reported SPD severity as well as psychosocial impairment associated with skin-picking. After the four-week training, the scores for the disorder-specific questionnaires were significantly lowered. This finding is noteworthy since the participants of the STT/PMR groups practiced on average for 22 days (15 min daily) leading to a practice time of five hours and 30 min. Considering this restricted training period as well as the chronicity of SPD symptoms (participants on average had had clinical symptoms for more than 12 years), the effects of the training are encouraging.

We need to mention the following limitations of the present investigation. We studied a group of female SPD patients. Therefore, the results cannot be generalized to other groups. The study design included an active control group (PMR) that only differed from STT concerning the type of stimulation: tensing/relaxing vs soft brushing of specific body regions. The two comparison groups (PMR, STT) were matched on all variables save the one of interest. However, a subsequent investigation should also include a waiting-list group (without training) so information on spontaneous symptom fluctuations can be obtained.

In the future, the app-assisted self-monitoring should include symptoms of comorbid disorders. In the present investigation, approximately one third of the participants had an additional diagnosis of an anxiety disorder (generalized anxiety disorder (GAD), panic disorder). For example, daily changes concerning GAD-related worrying might influence the training and consequently its effectiveness.

The present study used a well-validated affective touch procedure: soft brushing of the skin. However, some studies have shown that skin-to-skin touch is processed differently in the brain, compared to soft bristle brushing. Direct skin contact more strongly recruits the insula and prefrontal cortex regions (Ebisch et al., 2014, Kress et al., 2011, Lindgren et al., 2012). Therefore, Soft Touch Training with skin contact (generated by self/ others) might enhance the effectiveness of the intervention. Further, adaptations of the training concerning duration/ frequency of sessions may also increase effectiveness of the training.

In conclusion, this study demonstrated that a brief app-assisted touch training was able to change the experience of touch from others and the associated brain activity/connectivity in those with SPD, as well as the symptom severity of SPD.

## Acknowledgment

This study was funded by the Austrian Science Fund (KLI 824).

## Declaration of Competing Interest

The authors declare that they have no known competing financial interests or personal relationships that could have appeared to influence the work reported in this paper.

## Data Availability

Data will be made available on request.
