# Supplementary material for: A neurobiological evaluation of soft touch training for patients with skin-picking disorder
Source: Neuroimage Clin. 2022 Nov 3;36:103254. doi: 10.1016/j.nicl.2022.103254 (PMC9668654; doi:10.1016/j.nicl.2022.103254)
Supplement: Supplementary data 1 [file mmc1.docx]

**Supplementary Table 1**. Descriptives (means, standard deviations) and F-statistics (F, df, p and part.η^2^**)** for the Touch experience during the MRI experiment.

| **Arousal** | | | | |
| --- | --- | --- | --- | --- |
| **Time** | **Type of touch** | **Group** | **M** | **SD** |
| Before the training | Nonaffective | STT | 4,1689 | 1,61382 |
|  |  | PMR | 4,3767 | 1,49948 |
|  |  | Total | 4,2654 | 1,55119 |
|  | Affective | STT | 3,5538 | 1,32768 |
|  |  | PMR | 3,0533 | 1,09375 |
|  |  | Total | 3,3214 | 1,23961 |
| After the training | Nonaffective | STT | 3,5667 | 1,45862 |
|  |  | PMR | 4,1624 | 1,68603 |
|  |  | Total | 3,8433 | 1,58235 |
|  | Affective | STT | 2,8528 | 1,39266 |
|  |  | PMR | 2,9295 | 1,03835 |
|  |  | Total | 2,8884 | 1,23053 |
| **ANOVA** |  |  |  |  |
| **effects** | **F (df)** | **P** | **part.η^2^** |  |
| „time“ | 5.63 (1,54) | .021 | .094 |  |
| „time*group“ | 3.24 (1,54) | .169 | .035 |  |
| „touch“ | 36.49 (1,54) | <.001 | .403 |  |
| „touch*group“ | 3.64 (1,54) | .062 | .063 |  |
| „time* touch“ | 0.001 (1,54) | .982 | <.001 |  |
| “Time* touch*group” | 0.28 (1,54) | .598 | .005 |  |
| “group” | 0.11 (1,54) | .737 | .002 |  |
|  |  |  |  |  |
| **Valence** | | | | |
| **Time** | **Type of touch** | **Group** | **M** | **SD** |
| Before the training | Nonaffective | STT | 3,7869 | 1,54536 |
|  |  | PMR | 4,0172 | 1,18863 |
|  |  | Total | 3,8938 | 1,38377 |
|  | Affective | STT | 5,8492 | 1,90765 |
|  |  | PMR | 6,6358 | 1,35423 |
|  |  | Total | 6,2144 | 1,70561 |
| After the training | Nonaffective | STT | 4,2096 | 1,72324 |
|  |  | PMR | 3,5865 | 1,24040 |
|  |  | Total | 3,9203 | 1,53735 |
|  | Affective | STT | 6,5722 | 1,67708 |
|  |  | PMR | 6,2013 | 1,35201 |
|  |  | Total | 6,4000 | 1,53256 |
| **ANOVA** |  |  |  |  |
| **effects** | **F (df)** | **p** | **part.η^2^** |  |
| „time“ | .323 (1,54) | .572 | .006 |  |
| „time*group“ | 16.60 (1,54) | <.001 | .235 |  |
| „touch“ | 95.53 (1,54) | <.001 | .639 |  |
| „touch*group“ | 0.67 (1,54) | .417 | .012 |  |
| „time* touch“ | 0.53 (1,54) | .470 | .010 |  |
| “Time*touch*group” | 0.56 (1,54) | .459 | .010 |  |
| “group” | 0.01 (1,54) | .984 | <.001 |  |
|  |  |  |  |  |
| **Urge to pick** | | | | |
| **Time** | **Type of touch** | **Group** | **M** | **SD** |
| Before the training | Nonaffective | STT | 3,7432 | 1,77217 |
|  |  | PMR | 4,0378 | 1,88124 |
|  |  | Total | 3,8800 | 1,81290 |
|  | Affective | STT | 3,4902 | 1,72519 |
|  |  | PMR | 2,8557 | 1,53241 |
|  |  | Total | 3,1956 | 1,65489 |
| After the training | Nonaffective | STT | 3,3950 | 1,73277 |
|  |  | PMR | 3,8301 | 1,72602 |
|  |  | Total | 3,5970 | 1,72779 |
|  | Affective | STT | 2,6806 | 1,31781 |
|  |  | PMR | 2,8301 | 1,10690 |
|  |  | Total | 2,7500 | 1,21584 |
| **ANOVA** |  |  |  |  |
| **effects** | **F (df)** | **p** | **part.η^2^** |  |
| „time“ | 3.55 (1, 54) | .065 | .062 |  |
| „time*group“ | 1.57A (1, 54) | .216 | .028 |  |
| „touch“ | 21.70 (1, 54) | <.001 | .287 |  |
| „touch*group“ | 3.23 (1, 54) | .078 | .056 |  |
| „time*type of touch“ | 0.36 (1, 54) | .554 | .007 |  |
| “Time*touch*group” | 1.88 (1, 54) | .176 | .034 |  |
| “group” | 0.03 (1, 54) | .856 | .001 |  |
|  |  |  |  |  |

Footnote: STT: soft Touch Training; PMR: Progressive Muscle Relaxation
