# Supplementary material for: A neurobiological evaluation of soft touch training for patients with skin-picking disorder
Source: Neuroimage Clin. 2022 Nov 3;36:103254. doi: 10.1016/j.nicl.2022.103254 (PMC9668654; doi:10.1016/j.nicl.2022.103254)
Supplement: Supplementary data 2 [file mmc2.docx]

| **Activity** |  |  |  |  |  |  |  |
| --- | --- | --- | --- | --- | --- | --- | --- |
| **Group comparison (STT, PMR) for the contrast affective vs. nonaffective touch, after vs. before the 4-week training** |  |  |  |  |  |  |  |
| **Contrast** | **ROI** | **H** | **X** | **Y** | **Z** | **t** | **P(FWE)** |
| STT_gt_PMR_affective_gt_nonaffective_post_gt_pre | Striatum | L | -21 | 6 | -12 | 2.88 | 0.2225 |
| STT_gt_PMR_affective_gt_nonaffective_post_gt_pre | Striatum | R | 15 | -3 | 24 | 1.74 | 0.8079 |
| STT_gt_PMR_affective_gt_nonaffective_post_gt_pre | PFC | L | -27 | 9 | -15 | 2.67 | 0.9385 |
| STT_gt_PMR_affective_gt_nonaffective_post_gt_pre | PFC | R | 12 | 15 | 63 | 3.14 | 0.6882 |
| STT_gt_PMR_affective_gt_nonaffective_post_gt_pre | Insula | L | -30 | 9 | -15 | 1.95 | 0.6366 |
| STT_gt_PMR_affective_gt_nonaffective_post_gt_pre | Insula | R | 39 | 0 | -18 | 1.76 | 0.7232 |
| STT_gt_PMR_affective_gt_nonaffective_post_gt_pre | Thalamus | L | -21 | -33 | 3 | 2.31 | 0.3841 |
| STT_gt_PMR_affective_gt_nonaffective_post_gt_pre | Thalamus | R | 3 | -21 | 12 | 1.68 | 0.6621 |
|  |  |  |  |  |  |  |  |
| PMR_gt_STT_affective_gt_nonaffective_post_gt_pre | PFC | L | -33 | 39 | 24 | 2.62 | 0.9529 |
| PMR_gt_STT_affective_gt_nonaffective_post_gt_pre | Insula | L | -39 | -15 | 3 | 2.17 | 0.5166 |
| PMR_gt_STT_affective_gt_nonaffective_post_gt_pre | Insula | R | 39 | -15 | -3 | 2.40 | 0.3886 |
| PMR_gt_STT_affective_gt_nonaffective_post_gt_pre | Parietal Operculum | L | -60 | -27 | 18 | 2.96 | 0.0764 |
| PMR_gt_STT_affective_gt_nonaffective_post_gt_pre | Thalamus | R | 15 | -12 | 3 | 1.70 | 0.6574 |
| PMR_gt_STT_affective_gt_nonaffective_post_gt_pre | SMG | L | -63 | -27 | 21 | 3.25 | 0.0590 |
| PMR_gt_STT_affective_gt_nonaffective_post_gt_pre | PFC | L | -33 | 39 | 24 | 2.62 | 0.9529 |
|  |  |  |  |  |  |  |  |
| **Group comparison (STT, PMR) for the contrast affective vs. nonaffective touch, before the 4-week training** |  |  |  |  |  |  |  |
| **Contrast** | **ROI** | **H** | **X** | **Y** | **Z** | **t** | **P(FWE)** |
| STT_gt_PMR_pre_affective_gt_nonaffective | Striatum | R | 24 | 3 | 12 | 1.73 | 0.7757 |
| STT_gt_PMR_pre_affective_gt_nonaffective | Insula | R | 36 | 0 | 12 | 2.00 | 0.5622 |
| STT_gt_PMR_pre_affective_gt_nonaffective | Parietal Operculum | R | 51 | -24 | 21 | 3.07 | 0.0515 |
| STT_gt_PMR_pre_affective_gt_nonaffective | SMG | R | 48 | -24 | 36 | 2.51 | 0.2023 |
|  |  |  |  |  |  |  |  |
| PMR_gt_STT_pre_affective_gt_nonaffective | Striatum | L | -21 | 9 | -12 | 2.34 | 0.4614 |
| PMR_gt_STT_pre_affective_gt_nonaffective | Striatum | R | 30 | -3 | -3 | 1.97 | 0.6613 |
| PMR_gt_STT_pre_affective_gt_nonaffective | PFC | R | 30 | 9 | 60 | 3.39 | 0.4289 |
| PMR_gt_STT_pre_affective_gt_nonaffective | Insula | L | -45 | 6 | -6 | 2.04 | 0.5428 |
| PMR_gt_STT_pre_affective_gt_nonaffective | Thalamus | L | -21 | -33 | 3 | 2.23 | 0.3794 |
| PMR_gt_STT_pre_affective_gt_nonaffective | SMG | L | -48 | -45 | 39 | 1.75 | 0.6356 |
| PMR_gt_STT_pre_affective_gt_nonaffective | SMG | R | 54 | -45 | 51 | 2.06 | 0.5066 |
|  |  |  |  |  |  |  |  |
| **Group comparison (STT, PMR) for the contrast affective vs. nonaffective touch, after the 4-week training** |  |  |  |  |  |  |  |
| **Contrast** | **ROI** | **H** | **X** | **Y** | **Z** | **t** | **P(FWE)** |
| STT_gt_PMR_post_affective_gt_nonaffective | Striatum | L | -9 | 6 | -9 | 2.46 | 0.4473 |
| STT_gt_PMR_post_affective_gt_nonaffective | Striatum | R | 15 | -3 | 24 | 2.02 | 0.6946 |
| STT_gt_PMR_post_affective_gt_nonaffective | PFC | R | 12 | 15 | 60 | 2.59 | 0.9726 |
| STT_gt_PMR_post_affective_gt_nonaffective | Thalamus | L | -12 | -36 | 3 | 2.15 | 0.4776 |
| STT_gt_PMR_post_affective_gt_nonaffective | Thalamus | R | 18 | -30 | 12 | 2.13 | 0.4731 |
|  |  |  |  |  |  |  |  |
| PMR_gt_STT_post_affective_gt_nonaffective | Striatum | L | -6 | 9 | 6 | 2.11 | 0.6590 |
| PMR_gt_STT_post_affective_gt_nonaffective | Striatum | R | 9 | 3 | 9 | 2.00 | 0.7015 |
| PMR_gt_STT_post_affective_gt_nonaffective | Insula | R | 39 | -15 | -3 | 2.62 | 0.2871 |
| PMR_gt_STT_post_affective_gt_nonaffective | Parietal Operculum | L | -63 | -24 | 15 | 2.39 | 0.2291 |
| PMR_gt_STT_post_affective_gt_nonaffective | Parietal Operculum | R | 60 | -24 | 15 | 3.05 | 0.0635 |
| PMR_gt_STT_post_affective_gt_nonaffective | Thalamus | R | 15 | -9 | 6 | 2.15 | 0.4610 |
| PMR_gt_STT_post_affective_gt_nonaffective | SMG | L | -63 | -24 | 21 | 2.70 | 0.1912 |
|  |  |  |  |  |  |  |  |
| **Within-group analyses for the contrast affective vs. nonaffective touch, after vs. before the 4-week training** |  |  |  |  |  |  |  |
| **Contrast** | **ROI** | **H** | **X** | **Y** | **Z** | **t** | **P(FWE)** |
| within_STT_affective_gt_nonaffective_postgtpre | Striatum | L | -18 | 6 | -12 | 2.25 | 0.57823 |
| within_STT_affective_gt_nonaffective_postgtpre | PFC | R | 6 | 21 | -12 | 2.49 | 0.98605 |
| within_STT_affective_gt_nonaffective_postgtpre | Thalamus | L | -21 | -33 | 3 | 2.35 | 0.37913 |
|  |  |  |  |  |  |  |  |
| within_STT_affective_gt_nonaffective_pregtpost | Striatum | L | -18 | 6 | 0 | 2.33 | 0.53059 |
| within_STT_affective_gt_nonaffective_pregtpost | Striatum | R | 24 | 3 | 12 | 2.68 | 0.33344 |
| within_STT_affective_gt_nonaffective_pregtpost | PFC | L | -39 | 30 | 27 | 3.72 | 0.37128 |
| within_STT_affective_gt_nonaffective_pregtpost | PFC | R | 27 | 36 | 24 | 3.66 | 0.42750 |
| within_STT_affective_gt_nonaffective_pregtpost | Insula | L | -33 | -27 | 9 | 2.58 | 0.32786 |
| within_STT_affective_gt_nonaffective_pregtpost | Insula | R | 36 | -21 | 6 | 3.39 | 0.08166 |
| within_STT_affective_gt_nonaffective_pregtpost | Thalamus | L | -15 | -6 | 9 | 2.28 | 0.40656 |
| within_STT_affective_gt_nonaffective_pregtpost | Parietal Operculum | L | -60 | -30 | 21 | 2.61 | 0.16773 |
| within_STT_affective_gt_nonaffective_pregtpost | Thalamus | R | 15 | -12 | 3 | 2.12 | 0.4656 |
| within_STT_affective_gt_nonaffective_pregtpost | SMG | L | -60 | -27 | 21 | 2.83 | 0.16614 |
|  |  |  |  |  |  |  |  |
|  |  |  |  |  |  |  |  |
| **Contrast** | **ROI** | **H** | **X** | **Y** | **Z** | **t** | **P(FWE)** |
| within_PMR_affective_gt_nonaffective_postgtpre | Insula | L | -42 | -12 | 3 | 2.37 | 0.50244 |
| within_PMR_affective_gt_nonaffective_postgtpre | Parietal Operculum | L | -63 | -27 | 18 | 2.06 | 0.41352 |
| within_PMR_affective_gt_nonaffective_postgtpre | Parietal Operculum | R | 54 | -21 | 15 | 2.40 | 0.27264 |
| within_PMR_affective_gt_nonaffective_postgtpre | SMG | L | -63 | -24 | 33 | 2.59 | 0.29806 |
| within_PMR_affective_gt_nonaffective_postgtpre | SMG | R | 63 | -21 | 21 | 2.19 | 0.43536 |
|  |  |  |  |  |  |  |  |
| within_PMR_affective_gt_nonaffective_pregtpost | Striatum | L | -21 | 6 | -12 | 2.18 | 0.69421 |
| within_PMR_affective_gt_nonaffective_pregtpost | Striatum | R | 27 | 0 | 0 | 2.79 | 0.35366 |
| within_PMR_affective_gt_nonaffective_pregtpost | PFC | L | -24 | 45 | -15 | 4.48 | 0.14823 |
| within_PMR_affective_gt_nonaffective_pregtpost | PFC | R | 21 | 51 | -12 | 3.43 | 0.71834 |
| within_PMR_affective_gt_nonaffective_pregtpost | Insula | L | -30 | 12 | -15 | 2.21 | 0.58501 |
| within_PMR_affective_gt_nonaffective_pregtpost | Insula | R | 27 | 18 | -12 | 2.17 | 0.59865 |
| within_PMR_affective_gt_nonaffective_pregtpost | Thalamus | R/L | 0 | -15 | 9 | 2.26 | 0.49313 |
| within_PMR_affective_gt_nonaffective_pregtpost | Parietal Operculum | L | -33 | -33 | 21 | 1.73 | 0.55158 |
| within_PMR_affective_gt_nonaffective_pregtpost | Thalamus | R | 3 | -18 | 12 | 2.45 | 0.39443 |
| within_PMR_affective_gt_nonaffective_pregtpost | SMG | L | -45 | -51 | 45 | 3.02 | 0.18702 |
| within_PMR_affective_gt_nonaffective_pregtpost | SMG | R | 51 | -45 | 51 | 2.04 | 0.64111 |
|  |  |  |  |  |  |  |  |
|  |  |  |  |  |  |  |  |
| **Connectivity** |  |  |  |  |  |  |  |
| **Seed: SMG** |  |  |  |  |  |  |  |
| **Contrast** | **ROI** | **H** | **X** | **Y** | **Z** | **t** | **P(FWE)** |
| STT_gt_PMR_postgtpre_affective_gt_nonaffective | Striatum | R | 30 | 0 | 0 | 1.75 | 0.86274 |
| STT_gt_PMR_postgtpre_affective_gt_nonaffective | PFC | R | 45 | 27 | 24 | 2.92 | 0.90946 |
| STT_gt_PMR_postgtpre_affective_gt_nonaffective | Thalamus | L | -12 | -12 | 0 | 2.31 | 0.45224 |
| STT_gt_PMR_postgtpre_affective_gt_nonaffective | Thalamus | R | 18 | -27 | 9 | 2.79 | 0.21473 |
|  |  |  |  |  |  |  |  |
| PMR_gt_STT_postgtpre_affective_gt_nonaffective | Striatum | L | -30 | -3 | -3 | 2.22 | 0.65514 |
| PMR_gt_STT_postgtpre_affective_gt_nonaffective | Striatum | R | 27 | -9 | -9 | 1.72 | 0.87118 |
| PMR_gt_STT_postgtpre_affective_gt_nonaffective | Insula | L | -42 | 9 | -12 | 1.83 | 0.76731 |
| PMR_gt_STT_postgtpre_affective_gt_nonaffective | Insula | R | 45 | 12 | -6 | 1.84 | 0.75496 |
| PMR_gt_STT_postgtpre_affective_gt_nonaffective | Thalamus | L | -6 | 0 | 0 | 2.34 | 0.43450 |
| PMR_gt_STT_postgtpre_affective_gt_nonaffective | Parietal Operculum | R | 63 | -30 | 21 | 1.73 | 0.55366 |

Footnote: STT: Soft Touch Training; PMR: Progressive Muscle Relaxation; gt: greater; PFC: prefrontal cortex; SMG: supramarginal gyrus
